# Health Tract, No. 25

**Published:** 1860-04

**Authors:** 


### HEALTH TRACT, No. 25.

### SLEEPING.

INABILITY to sleep is the first step toward madness, while sound and sufficient sleep imparts a vigor to the mind, and a feeling of wellness and activity to the body, which are beyond price. To be able to go to sleep within a few minutes of reaching the pillow, and to sleep soundly until the morning breaks, and to do this for weeks and months together, is perfectly delightful. How such a thing may be brought about, and kept up, as a general rule, is certainly well worth knowing, and will be appreciated, even by those who have lost but half a night's sleep. The reader can study out the reasons of the suggestions at his leisure.

Both in city and country the chamber should be on the second, third or higher floor; its windows should face the east or south, so as to have the drying and purifying influences of the blessed sunlight; there should be no curtains to the bed or windows, nor should there be any hanging garments or other woven fabrics except the clothes worn during the day, each article of which should be spread out by itself, for the purpose of thorough airing. There should be no carpet on the floor of a sleeping-room, except a single strip by the side of the bed, to prevent a sudden shock by the warm foot coming in contact with a cold floor. Carpets collect dust and dirt and filth and dampness, and are the invention of laziness to save labor and hide uncleanness.

Ordinarily, mattresses of shucks, chaff, straw, or curled hair are best to sleep upon. For old persons and those of feeble vitality, there is nothing better than a clean feather bed. No one can sleep well if cold. Have as little covering as possible from just above the knees upwards, but cover the legs and feet abundantly, for by keeping them warm, the blood is withdrawn from the brain, and to that extent, dreaming is prevented.

There should be no standing fluid of any description, nor a particle of food or vegetation or any decayable substance allowed to remain in a bed-room for a moment; nor should any light be kept burning, except from necessity, as all these things corrupt the air which is breathed while sleeping.

The entire furniture of a chamber should be the bed, two or three wooden chairs, a table and a bureau or chest of drawers. Every article of bed-clothing should be thrown over a chair or table by itself, and the mattress remain exposed, until the middle of the afternoon; not later, lest the damps of the evening should impregnate them. From morning until afternoon of every sunshiny day, the windows of the chamber should be hoisted fully. The fire-place should be kept open, at least during the night, thus affording a draft from the crevices of doors and windows. As foul air is lightest in warm weather, it is best that the sash should be let down at the top half an inch or more, and the lower one elevated several inches; by this means the pure and cool air from without enters and drives the heated impure air upwards and outwards.

In a very cold room, without a good draught or ventilation, carbonic acid being generated by the sleeper, becomes heavy and falls to the floor; this gas has no nourishment for the lungs, and to breathe it wholly for two minutes, is to die; it is this which causes suffocation in descending some wells. In summer it goes to the ceiling, in winter to the floor; hence it is more important that a sleeping-room should have a very gentle current of air in winter than in summer.

Never go to bed with cold or damp feet, else refreshing sleep is impossible; but spend the last five or ten minutes before bed-time, at least in firetime of year, in drying and heating the feet before the fire, with the stockings off. Indians and hunters sleep with their feet towards the camp-fire.

Different persons require different amounts of sleep, according to age, sex, and occupation. Nature must make the apportionment, and will always do it wisely and safely; and there is only one method of doing it. Do not sleep a moment in the day, or if essential do not exceed ten minutes, for this will refresh more than if you sleep an hour, or longer. Go to bed at a regular early hour, not later than ten, and get up as soon as you wake of yourself in the morning; follow this up for a week or two, and if there is no actual disease, nature will always arouse the sleeper as soon as enough sleep has been taken to repair the expenditures of the preceding day, a little more or less in proportion to the amount of bodily and mental effort made the day before. Commonly there will be but a few minutes' difference for weeks together. It is not absolutely necessary to get up and dress, but only to avoid a second nap. Sometimes it is advantageous to remain in bed until the feeling of tiredness, with which most persons are familiar, has passed from the limbs. It is safest and best for all to take breakfast before going out of doors in the morning, whether in summer or winter, most especially in new, flat or damp countries, as a preventive of chill and fever.

If from any cause you get up during the night, throw open the bed-clothes, so as to give the bedding an airing, and also with the hands give the whole body a good rubbing for a minute or two; the effect will be an immediate feeling of refreshment, and a more speedy falling to sleep again. This was Franklin's remedy in case of restlessness at night.

When it is remembered that one third of our whole time is spent in our chambers, and that only uncorrupted air can complete the process of digestion and assimilation and purify the blood, it is most apparent that the utmost pains should be taken to secure the breathing of a pure atmosphere during the hours of sleep; and that the most diligent attention in this regard is indispensable to high health.

---

### HEALTH WITHOUT MEDICINE OR MONEY.

A Medical Library which never advises a dose of medicine, except in cholera, may be found in the following works, written by Dr. W. W. Hall, of 42 Irving Place, New-York, after having spent many years in special and exclusive attention to diseases of the throat and lungs:

Hall's Journal of Health, six volumes, $1.25 each: whole set, ..........$7.00
Health and Disease, a Book for the People, third edition, 298 pages, ...... 1.00
Bronchitis and Kindred Diseases, ninth edition, 382 pages,................ 1.00
Consumption, second edition, 280 pages, 1859, ............................ 1.00

The object of these books is to show, to the young especially, how health may be preserved by natural agencies, and how, by the same means, to remedy ordinary ailments, such as cold feet, sick-headache, constipation, neuralgia, dyspepsia, etc.

Hall's Journal of Health is published monthly, for one dollar a year, specimen numbers ten cents.

The Fireside Monthly is $1.50 a year, specimens twelve cents; it excludes fiction, and is devoted to science, literature, and practical life. This, with the Journal of Health, will be sent for two dollars a year.

The Health Tracts are furnished at 25 cents a hundred, assorted.

